# Long-term clinical outcomes in patients with untreated non-culprit intermediate coronary lesion and evaluation of predictors by using virtual histology-intravascular ultrasound; a prospective cohort study

**DOI:** 10.1186/s12872-019-1173-5

**Published:** 2019-08-05

**Authors:** Young Hoon Seo, Yong-Kyun Kim, In Geol Song, Ki-Hong Kim, Taek-Geun Kwon, Jang-Ho Bae

**Affiliations:** Division of Cardiology, Heart Center, Konyang University Hospital, 158, Gwanjeodong-Ro, Seo-Gu, Daejeon, 35365 South Korea

**Keywords:** Coronary artery disease, Ultrasonography, interventional, Atherosclerosis, Coronary angiography, Myocardial ischemia

## Abstract

**Background:**

It is uncertain whether the coronary lesion with intermediate stenosis is more likely to cause cardiovascular events than a normal or minimal lesion. We conducted a single-center, prospective cohort study to identify long-term clinical outcomes of patients with untreated non-culprit intermediate lesion and evaluate its predictor of cardiovascular events by using virtual histology-intravascular ultrasound (VH-IVUS).

**Methods:**

Subjects with non-culprit intermediate lesion underwent VH-IVUS were prospectively registered after percutaneous coronary intervention at the culprit lesion. Intermediate lesion was defined as 30 to 70% stenosis in coronary angiography and primary outcome was an occurrence of major adverse cardiovascular events (MACE) defined as all-cause death, intermediate lesion revascularization (InLR), minimal lesion revascularization (MnLR, unplanned revascularization elsewhere in the target vessel or in other coronary arteries which looked normal or minimal stenosis), cerebrovascular events, or non-fatal myocardial infarction (MI). The mean follow-up period was 4.2 years.

**Results:**

Total 25 MACE, approximately 7% incidence annually, were identified during a follow-up period in 86 patients with 89 intermediate lesions. InLR (*n* = 13) was a most common event followed by MnLR (*n* = 6), non-fatal MI (*n* = 4), all-cause death (*n* = 3), and cerebrovascular events (n = 1). Diameter stenosis (OR 1.07, 95% CI 1.01–1.12, *p* = 0.015), plaque burden (PB, OR 1.07, 95% CI 1.00–1.15, *p* = 0.040), fibrofatty area (FFA, OR 1.61, 95% CI 1.10–2.38, *p* = 0.016), PB ≥ 70% (OR 3.93, 95% CI 1.28–12.07, *p* = 0.018), and area stenosis ≥ 50% (OR 2.94, 95% CI 1.01–8.56, *p* = 0.042) showed significant relationships with an occurrence of MACE. In multivariable Cox-proportional hazard analysis, FFA in intermediate lesion was an only independent predictor of MACE (HR 1.36, 95% CI 1.05–1.77, *p* = 0.019).

**Conclusions:**

Untreated intermediate lesions had a significantly higher chance for requiring revascularization compared with a normal or minimal lesion. And also, a large FFA in intermediate lesion was a significant predictor of cardiovascular events and which finding was mainly driven by coronary-related events, in particularly intermediate lesion progression.

## Background

Virtual histology-intravascular ultrasound (VH-IVUS) and fractional flow reserve (FFR) are the most widely used methods to decide the need for revascularization in intermediate coronary lesion. Although both modalities showed comparable results in intermediate lesion [1], there is a difference in clinical information we can identify from each study. While FFR is a valuable tool to verify the hemodynamic significance in specific lesion, VH-IVUS has an ability to assess the anatomical feature in coronary lesion and thus can detect high-risk morphologic characteristics. Grayscale IVUS provides anatomical information such as plaque morphology, plaque burden (PB), luminal area or diameter, area stenosis (AS), et al. In the era of VH-IVUS, more information can be obtained by clarifying the plaque composition and detecting the high-risk plaque such as thin-cap fibroatheroma (TCFA), a kind of vulnerable plaque which prone to thrombosis with or without rupture and at risk for rapid progression [2, 3].

The PROSPECT study showed that the VH-TCFA in non-culprit lesion is one of the significant predictors of cardiovascular events in patients with acute coronary syndrome (ACS) [4]. The VIVA study [5] and the ATHEROREMO-IVUS sub-study [6] confirmed that VH-TCFA in non-culprit lesion is an independent predictor of cardiovascular events in patients with coronary artery disease (CAD) including stable ischemic heart disease (SIHD). However, although plaque rupture is common cause of cardiovascular events, not all plaque ruptures occur from VH-TCFA [7] and approximately one-third in patients with cardiovascular events had another pathophysiology rather than a plaque rupture [3, 8]. It is unclear which plaque composition of non-culprit lesion is associated with the future cardiovascular events.

Little is known about the natural history of untreated intermediate coronary lesion and its plaque characteristics affecting clinical outcomes. And also, in non-culprit lesion, whether lesion showing intermediate stenosis has a higher chance for cause future cardiovascular events than a normal or minimal lesion is still unknown. We assessed long-term clinical outcomes of patients with untreated non-culprit intermediate lesion and evaluated its plaque characteristics affecting cardiovascular events by using VH-IVUS.

## Methods

### Study design, population, and primary outcome

A single-center, prospective cohort study was conducted at Heart Center, Konyang University Hospital, Daejeon, South Korea. Among patients underwent coronary angiography (CAG) with ischemic heart disease, subjects with non-culprit intermediate lesion were enrolled in the study after successful percutaneous coronary intervention (PCI) at the culprit lesion. Intermediate lesions were analyzed by VH-IVUS after PCI during a same procedure and patients’ medical records, laboratory findings, angiographic and VH-IVUS findings were obtained. Exclusion criteria were as follows; 1) patient younger than 20 year, 2) cardiac arrest, 3) history of coronary artery bypass graft (CABG) and 4) multi-vessel disease that culprit or non-culprit lesions were not clearly identified. Subjects have included for 2 years and additionally followed-up for 3.5 years after completion of inclusion.

Intermediate lesion was defined as 30 to 70% stenosis in baseline CAG. Primary outcome was occurrence of major adverse cardiovascular events (MACE), which consisted of all-cause death, intermediate lesion revascularization (InLR, PCI or CABG at enrolled intermediate lesion), minimal lesion revascularization (MnLR, unplanned revascularization elsewhere in the target vessel or in other coronary arteries which looked normal or stenosis less than 30%), cerebrovascular event (intracranial hemorrhage, stroke or transient ischemia attack), and non-fatal myocardial infarction (MI). Revascularization was conducted in the case of ACS or recurrent angina despite of best medical therapy. Culprit lesion revascularization was not included in primary outcome unless which causes fatal or non-fatal MI.

If a patient experienced two or more MACE at same time or sequentially, it counted as one incidence of MACE and time-to-event duration was defined as duration from enrollment to first event. The study was approved by the Institutional Review Board of Konyang University Hospital and performed in accordance with the criteria described in the declaration of Helsinki. Written informed consent was obtained from all subjects.

### IVUS examination and spectral analysis of radiofrequency data

The VH-IVUS examination was performed with a dedicated VH-IVUS console (Volcano Therapeutics, Rancho Cordova, California) during the CAG after intracoronary administration of 100 to 200 μg nitroglycerin. A 20-MHz, 2.9F monorail, electronic Eagle Eye Gold IVUS catheter (Volcano Therapeutics, Rancho Cordova, California) was advanced into the target lesion and automatic pullback at 0.5 mm/s was done. The VH-IVUS image was recorded on a DVD-ROM for offline analysis later. The VH-IVUS uses spectral analysis of IVUS radiofrequency data to construct a tissue map.

Qualitative and quantitative analyses of grayscale IVUS images were performed according to the criteria of the American College of Cardiology’s Clinical Expert Consensus Document on IVUS [9]. We analyzed plaque composition of cross sectional image obtained from minimal luminal area site. Reference segments were analyzed similar to the intermediate lesion. The proximal and distal reference segments were the most normal-looking cross sections ≤10 mm distal and proximal to the intermediate lesion. Reference data were the average of the proximal and distal reference segments.

**Fig. 1:**
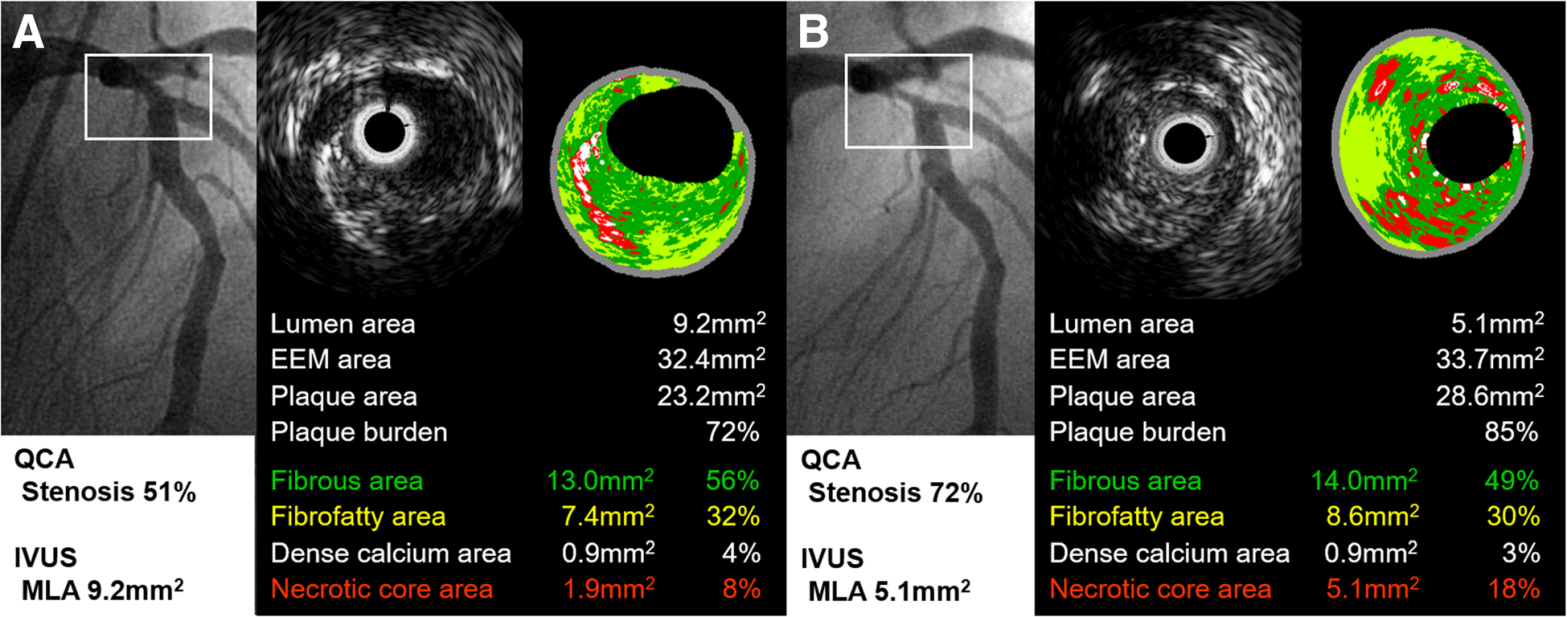
Angiographic and VH-IVUS images of the study. Figure represents angiographic and VH-IVUS images of intermediate lesion in 74-year-old male patient who underwent PCI at the culprit lesion, mid segment of left circumflex artery (not shown). Proximal segment of left anterior descending artery was enrolled to the study. **a** Intermediate lesion in baseline angiography (white square). **b** After 9 months, lesion progression caused recurrent angina and was so treated with PCI. VH-IVUS, virtual histology-intravascular ultrasound; PCI, percutaneous coronary intervention; QCA, quantitative coronary angiography; MLA, minimal luminal area; EEM, external elastic membrane

Spectral analysis of IVUS was done on the intermediate lesion with customized software (IVUS Lab; Volcano Therapeutics, Rancho Cordova, California) by two examiners who were unaware of clinical characteristics of the patients. For both the lumen and the media-adventitia interface, automatic border detection was done at the predefined lesion segment. Then, the border detection was manually corrected again in the lesion after automatic border detection. After confirming the border detection, the software automatically calculates and shows the results. For each frame, histologic findings were expressed in colors, such as green for fibrous, green-yellow for fibrofatty, white for dense calcium and red for necrotic core area (Fig. 1). The plaque volume of entire lesion and the percentage of each component were displayed also [10–12].

### Statistical analysis

The statistical data was processed using SPSS program (version 18.0, USA) and *p*-value less than 0.05 was considered statistically significant. Patient demographics and lesion characteristics were compared according to the occurrence of MACE. Chi-square test or Fisher’s exact test was done for analyzing categorical variables and independent t-test was done for analyzing continuous variables. The proportion of individual or total MACE was expressed based on with patient.

Analyses to see the univariate predictor of MACE were done for variables which showed statistical significance in lesion characteristics or which were well-demonstrated in previously published studies. Binary logistic regression analysis was done when variable was continuous. Multivariable Cox-proportional hazard analysis was done to demonstrate the independent predictors of MACE. Clinical relevant and factors showed significant relationship with MACE in univariate analysis with *p* value less than 0.05 were entered into the multivariable analysis.

## Results

### Demographics and clinical outcomes

Total 96 consecutive patients with 100 intermediate lesions were included in the study. After excluding subjects lost to follow-up, 86 patients with 89 lesions completely followed-up during mean 50.7 ± 5.7 months. Patient- and lesion-level characteristics were compared according to the occurrence of MACE. There were no significant differences in baseline patient demographics between MACE group (*n* = 25, 29.1%) and no MACE group (*n* = 61, 70.9%) (Table 1). Baseline lesion analysis showed that diameter stenosis (DS, 47.6 ± 8.1% vs. 42.2 ± 1.2%, *p* = 0.012) measured with quantitative coronary angiography, PB (66.0 ± 8.6% vs. 61.6 ± 9.9%, *p* = 0.045) and fibrofatty area (FFA, 1.5 ± 1.8 mm^2^ vs. 0.8 ± 0.8 mm^2^, *p* = 0.047) were larger in MACE group (*n* = 27, 30.3%) than no MACE group (*n* = 62, 69.7%). There were no other significant differences in angiographic or VH-IVUS findings between two groups (Table 2).

**Table 1:** Baseline patient demographics

Variables	MACE *n* = 25 (29.1%)	no MACE *n* = 61 (70.9%)	*P* value
Age, y	59.3 ± 12.0	62.5 ± 12.2	0.274
Male, n (%)	16 (64.0)	46 (75.4)	0.284
Diagnosis, n (%)			0.223
SAP	10 (40.0)	38 (62.3)	
UA	2 (8.0)	4 (6.6)	
NSTEMI	5 (20.0)	6 (9.8)	
STEMI	8 (32.0)	13 (21.3)	
Ejection fraction, %	67.2 ± 10.4	64.2 ± 11.2	0.280
Hypertension, n (%)	12 (48.0)	27 (44.3)	0.752
Diabetes mellitus, n (%)	8 (32.0)	15 (24.6)	0.481
Smoking, n (%)	11 (44.0)	22 (36.1)	0.492
Dyslipidemia, n (%)	10 (41.7)	26 (43.3)	0.889
Prior myocardial infarction, n (%)	3 (12.0)	2 (3.3)	0.145
Lipid profile			
Total cholesterol, mg/dL	190.5 ± 55.0	204.2 ± 82.0	0.458
Triglyceride, mg/dL	180.1 ± 124.9	183.5 ± 226.2	0.947
HDL cholesterol, mg/dL	43.7 ± 9.2	43.6 ± 11.1	0.967
LDL cholesterol, mg/dL	118.2 ± 41.3	128.1 ± 32.7	0.261
High sensitivity C-reactive protein, mg/L	0.2 ± 0.2	0.6 ± 1.4	0.051
Prescribed medication, %			
Aspirin	100	100	
P2Y12 inhibitor	100	100	
Beta blocker	64.0	70.0	0.588
Calcium channel blocker	16.0	28.3	0.230
ACEi	60.0	46.7	0.263
ARB	24.0	15.0	0.357
Lipid lowering agent	80.0	81.7	0.595

*MACE* major adverse cardiovascular events, *SAP* stable angina pectoris, *UA* unstable angina, *NSTEMI* non ST-segment elevation myocardial infarction, *STEMI* ST-segment elevation myocardial infarction, *HDL* high-density lipoprotein, *LDL* low-density lipoprotein, *ACEi* angiotensin-converting enzyme inhibitor, *ARB* angiotensin II receptor blocker

**Table 2:** Baseline angiographic and VH-IVUS findings of the intermediate lesions

Variables	MACE *n* = 27 (30.3%)	no MACE *n* = 62 (69.7%)	*P* value
Location			0.871
LM, n (%)	2 (7.4)	8 (12.9)	
LAD, n (%)	16 (59.3)	30 (48.4)	
LCX, n (%)	4 (14.8)	13 (21.0)	
RCA, n (%)	5 (18.5)	10 (16.1)	
Ramus intermedius, n (%)	0 (0)	1 (1.6)	
Diameter stenosis, % (QCA)	47.6 ± 8.1	42.2 ± 1.2	0.012
Grayscale IVUS			
Diameter stenosis, %	29.1 ± 10.9	26.7 ± 8.1	0.314
Lesion length, mm	13.1 ± 6.6	13.9 ± 7.7	0.609
Area stenosis, %	39.2 ± 16.7	35.7 ± 13.7	0.321
Minimal luminal area, mm^2^	4.9 ± 1.6	5.8 ± 2.5	0.073
Minimal luminal diameter, mm	2.3 ± 0.3	2.4 ± 0.5	0.130
Plaque burden, %	66.0 ± 8.6	61.6 ± 9.9	0.045
Reference area, mm^2^	8.4 ± 2.6	8.6 ± 3.2	0.806
Lumen volume, mm^3^	92.9 ± 43.3	99.3 ± 57.0	0.607
Plaque volume, mm^3^	119.8 ± 94.1	114.7 ± 76.8	0.790
Remodeling index	1.0 ± 0.1	1.0 ± 0.1	0.814
VH-IVUS			
TCFA, n (%)	7 (25.9)	19 (30.6)	0.653
Fibrous area, mm^2^	4.4 ± 2.5	4.0 ± 2.3	0.472
Fibrofatty area, mm^2^	1.5 ± 1.8	0.8 ± 0.8	0.047
Dense calcium area, mm^2^	0.5 ± 0.5	0.5 ± 0.5	0.547
Necrotic core area, mm^2^	1.2 ± 1.2	1.1 ± 0.9	0.774
Percent fibrous area, %	59.0 ± 13.9	61.2 ± 14.2	0.511
Percent fibrofatty area, %	15.6 ± 13.1	11.0 ± 7.8	0.101
Percent dense calcium area, %	7.6 ± 6.4	9.3 ± 9.3	0.394
Percent necrotic core area, %	17.9 ± 14.0	18.4 ± 10.7	0.845
Fibrous volume, mm^3^	44.2 ± 46.6	42.7 ± 34.5	0.866
Fibrofatty volume, mm^3^	13.2 ± 18.0	8.8 ± 11.2	0.166
Dense calcium volume, mm^3^	5.5 ± 6.0	6.2 ± 6.2	0.639
Necrotic core volume, mm^3^	11.1 ± 10.4	11.8 ± 9.7	0.784
Percent fibrous volume, %	61.2 ± 12.1	61.0 ± 11.5	0.966
Percent fibrofatty volume, %	14.9 ± 9.6	11.7 ± 7.4	0.093
Percent dense calcium volume, %	7.9 ± 6.8	9.3 ± 7.6	0.413
Percent necrotic core volume, %	16.0 ± 10.4	17.9 ± 9.7	0.400

*VH-IVUS* virtual histology-intravascular ultrasound, *MACE* major adverse cardiovascular events, *LM* left main coronary artery, *LAD* left anterior descending artery, *LCX* left circumflex artery, *RCA* right coronary artery, *QCA* quantitative coronary angiography, *TCFA* thin-cap fibroatheroma

**Fig. 2:**
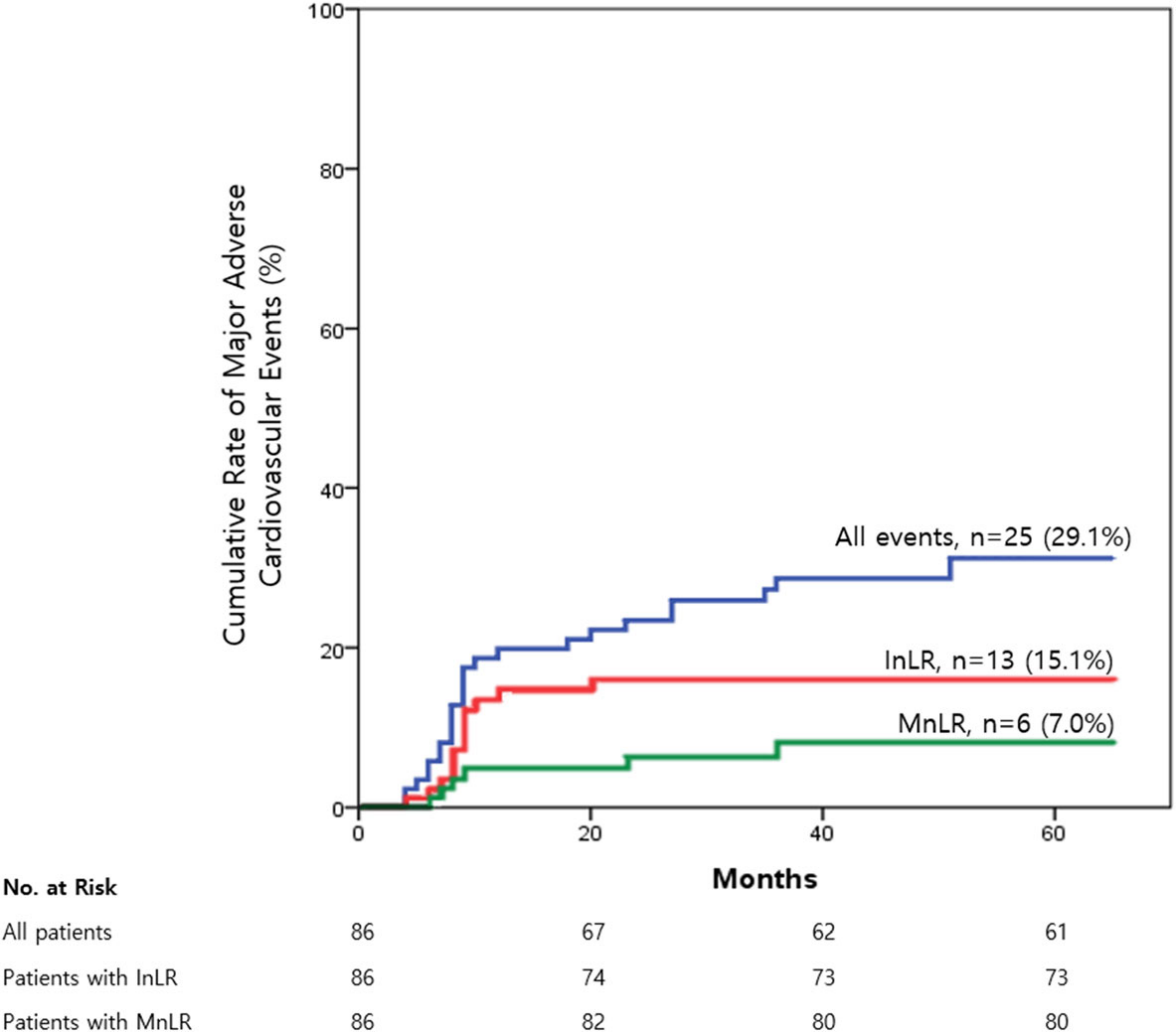
Cumulative incidence of primary outcome. Kaplan-Meier analysis showing cumulative incidence of all events, intermediate lesion revascularization, and minimal lesion revascularization as representative primary outcomes. Others were not shown in Figure because of small number of incidences. InLR, intermediate lesion revascularization; MnLR, minimal lesion revascularization

Total 25 MACE (from 25 patients with 27 intermediate lesions) were identified during a follow-up period and mean time-to-event duration was 14.4 ± 11.7 months. Minimal and maximal time-to-event duration was 4 months and 51 months, respectively. InLR (*n* = 13) was the most common event, followed by MnLR (*n* = 6), non-fatal MI (*n* = 4), all-cause death (*n* = 3) and cerebrovascular event (n = 1) (Table 3). One patient died the day after InLR and another patient underwent MnLR for non-fatal MI. These 2 cases were counted as 1 occurrence of MACE. Other 3 cases of non-fatal MI were developed from previously PCI-treated lesion. Cumulative incidence of composite outcomes, InLR, and MnLR along the follow-up duration is shown in Fig. 2**.**

**Table 3:** Incidence of MACE

Variables	Incidence	Proportion (*n* = 86)
All-cause death	3	3.5%
Intermediate lesion revascularization	13	15.1%
Minimal lesion revascularization	6	7.0%
Cerebrovascular event	1	1.2%
Non-fatal myocardial infarction	4	4.7%
Total	25	29.1%

*MACE* major adverse cardiovascular events

### Associations between IVUS findings and clinical outcomes

Results of univariate analysis for predictor of MACE are shown in Table 4. DS (Odds ratio, OR 1.07, 95% Confidence interval, CI 1.01–1.12, *p* = 0.015), PB (OR 1.07, 95% CI 1.00–1.15, *p* = 0.040), FFA (OR 1.61, 95% CI 1.10–2.38, *p* = 0.016), PB ≥ 70% (OR 3.93, 95% CI 1.28–12.07, *p* = 0.018) and AS ≥ 50% (OR 2.94, 95% CI 1.01–8.56, *p* = 0.042) were significantly related with an occurrence of MACE.

**Table 4:** Univariate analysis for the predictors of MACE

Variable	Odds ratio	95% Confidence interval	*P* value
Diameter stenosis (QCA)	1.07	1.01–1.12	0.015
Plaque burden	1.07	1.00–1.15	0.040
Fibrofatty area	1.61	1.10–2.38	0.016
Minimal luminal area < 4.0 mm^2^	1.69	0.64–4.45	0.284
Virtual histology-Thin-cap fibroatheroma	0.79	0.29–2.19	0.653
Plaque burden ≥ 70%	3.93	1.28–12.07	0.018
Area stenosis ≥ 50%	2.94	1.01–8.56	0.042

*MACE* major adverse cardiovascular events, *QCA* quantitative coronary angiography

Table 5 represents the associations between VH-IVUS findings and MACE using multivariable Cox-proportional hazard analysis. DS (Hazards ratio, HR 1.04, 95% CI 1.00–1.09, *p* = 0.030), FFA (HR 1.42, 95% CI 1.10–1.85, *p* = 0.008), PB ≥ 70% (HR 2.34, 95% CI 1.02–5.37, *p* = 0.046) and AS ≥ 50% (HR 2.59, 95% CI 1.15–5.84, *p* = 0.022) showed significant relationships with MACE in adjusted model (Model 1). In multivariable analysis (Model 2), only FFA was independently associated with the occurrence of MACE (HR 1.36, 95% CI 1.05–1.77, *p* = 0.019).

**Table 5:** Adjusted and multivariable analyses for the predictors of MACE

Variables	Model 1 - adjusted model			Model 2 - multivariable model		
	HR	95% CI	*P* value	HR	95% CI	*P* value
Diameter stenosis (QCA)	1.04	1.00–1.09	0.030	NS		
Plaque burden	1.05	1.00–1.12	0.070	NS		
Fibrofatty area	1.42	1.10–1.85	0.008	1.36	1.05–1.77	0.019
Plaque burden ≥ 70%	2.34	1.02–5.37	0.046	NS		
Area stenosis ≥ 50%	2.59	1.15–5.84	0.022	NS		

Model 1 – age, gender, hypertension, diabetes mellitus, smoking and dyslipidemia were adjusted

Model 2 – diameter stenosis, plaque burden, fibrofatty area, plaque burden ≥ 70% and area stenosis ≥ 50% were added in Model 1

*MACE* major adverse cardiovascular events, *HR* hazards ratio, *CI* confidence interval, *QCA* quantitative coronary angiography, *NS* non-significant

## Discussion

The main findings of present study are two things. First, in non-culprit coronary lesions, intermediate lesion had a significantly higher chance for requiring revascularization compared with a normal or minimal lesion. Second, a large FFA of intermediate lesion, validated by VH-IVUS, was significantly associated with the cardiovascular events mainly driven by coronary-related events, in particularly intermediate lesion progression.

Two landmark studies suggested that most cardiovascular events, especially acute MI, developed from mild or moderate stenosis [13, 14]. However, after that, many studies reported coronary artery stenosis was associated with acute coronary events although there were some differences in study designs [4–6, 15]. It is debated whether severity of stenosis is statistically associated with more cardiovascular events. So we assessed the natural history of intermediate lesions and evaluated that untreated intermediate lesion is more likely to cause cardiovascular events compared with normal or minimal stenosis.

Previous studies revealed that various parameters of grayscale IVUS [15–17] or VH-IVUS [18–20] were related with clinical features. Associations between fibrofatty plaque and unfavorable lesion characteristics or cardiovascular events were already reported [7, 21–23], but little is known regarding the long-term clinical outcomes. The fibrofatty plaque, a lipid-rich area related with positive arterial remodeling [21], may represent earlier than necrotic core in atherosclerotic progression. Kim et al. [7] reported that fibrofatty plaque may be another form of vulnerable plaque and Vazquez-Figueroa et al. [24] reported that fibrofatty plaque of intermediate lesion was significant finding for 1-year outcomes, which is consistent with our finding. That is, large FFA may be one of the features of high-risk plaque.

In our study, VH-TCFA was not a predictor of MACE. Although VH-TCFA is a well demonstrated poor prognostic factor, there are some factors causing result discrepancy between previous studies and present study. First, because of its non-objective definition, a confluent necrotic core in contact with the lumen [25], there can be an inaccurate measurement despite of physician’s caution. Second, VH-IVUS has low accuracy of detecting power for in vivo necrotic core [26], which is a most significant factor for defining the VH-TCFA. Third, each study has a different primary outcome which naturally results different predictor in statistical analysis. These might be the reasons explaining the discrepancy of our study from previous studies. And previous two reasons also impact that careful interpretation should be given in the study results relying on the necrotic core or VH-TCFA, therefore, detecting the high-risk plaque other than necrotic core or VH-TCFA is clinically important.

In the present study, high-sensitivity C-reactive protein (hs-CRP) showed no prognostic power on clinical outcomes unlike previous studies [27, 28]. All subjects of this study were enrolled after coronary event and hs-CRP was obtained just before or after the PCI, so this point may have caused the discrepancy.

Deciding to perform PCI in intermediate lesion is still difficult although VH-IVUS and FFR are established modalities for assessing the anatomical and physiological coronary stenosis. The PROSPECT, a large landmark study, showed that non-culprit lesions have a similar prevalence of future MACE in compared with those of culprit lesions [4]. Thus detecting the high-risk plaque in the non-culprit intermediate lesion will be useful way to predict and prevent future cardiovascular events. Our study showed that a large FFA of intermediate lesion was associated with poor long-term outcomes and thus can be considered as a feature of high-risk plaque.

Small number of subjects and relatively high proportion of patients lost to follow-up are major limitations of the study. Our study did not include culprit lesion-related events as primary outcomes because initially treated lesion-related events, in-stent restenosis or stent thrombosis, have another mechanism compared with the native lesion-derived cardiovascular events. Advantage of this study is that we included all eligible patients regardless of history of prior MI or presence of left main disease. More large, well-designed trials will be mandatory for further evaluation of the relationship between clinical outcomes and plaque characteristics of intermediate coronary lesions.

## Conclusions

Untreated intermediate lesions had a significantly higher chance for requiring revascularization compared with a normal or minimal lesion. And also, a large FFA in intermediate lesion was a significant predictor of cardiovascular events and which finding was mainly driven by coronary-related events, in particularly intermediate lesion progression.

## Data Availability

The datasets used and/or analysed during the current study are available from the corresponding author on reasonable request.

## Abbreviations

ACS: Acute coronary syndrome
AS: Area stenosis
CABG: Coronary artery bypass graft
CAD: Coronary artery disease
CAG: Coronary angiography
CI: Confidence interval
DS: Diameter stenosis
FFA: Fibrofatty area
FFR: Fractional flow reserve
HR: Hazards ratio
hs-CRP: High-sensitivity C-reactive protein
InLR: Intermediate lesion revascularization
MACE: Major adverse cardiovascular events
MI: Myocardial infarction
MnLR: Minimal lesion revascularization
OR: Odds ratio
PB: Plaque burden
PCI: Percutaneous coronary intervention
SIHD: Stable ischemic heart disease
TCFA: Thin-cap fibroatheroma
VH-IVUS: Virtual histology-intravascular ultrasound
